# Sub-inhibitory concentrations of tigecycline could attenuate the virulence of *Staphylococcus aureus* by inhibiting the product of α-toxin

**DOI:** 10.1128/spectrum.01344-24

**Published:** 2025-03-19

**Authors:** Junhong Shi, Li Shen, Yanghua Xiao, Cailing Wan, Bingjie Wang, Peiyao Zhou, Jiao Zhang, Weihua Han, Fangyou Yu

**Affiliations:** 1 Department of Clinical Laboratory, Shanghai Pulmonary Hospital, School of Medicine, Tongji University, Shanghai, China; Institute of Hydrobiology, CAS, Wuhan, China

**Keywords:** *Staphylococcus aureus*, tigecycline, virulence, α-toxin, SaeRS two-component system, host immune response

## Abstract

*Staphylococcus aureus* (*S. aureus*) infection is a serious threat to global health. This study aimed to investigate the anti-virulence efficacy of tigecycline against *S. aureus*. We used highly virulent *S. aureus* strains SA75 and JP30 to evaluate the effect of tigecycline on virulence, both of them isolated from the clinic. The MIC value of tigecycline against SA75 was 0.125 µg/mL, and that against JP30 was 0.25 µg/mL. Tigecycline did not affect the growth ability of bacteria at 0.015 µg/mL. Thus, subsequent discussions will focus on the effect of antibiotics at the latter subinhibitory concentrations that did not affect growth. First, the sub-MICs of tigecycline not only enhanced the sensitivity of *S. aureus* to oxidants and human whole blood but also weakened the hemolytic activity and cell adhesion level of *S. aureus*. Second, it undermined the survival of *S. aureus* in RAW264.7 and attenuated the macrophage inflammatory response induced by *S. aureus*. On the contrary, tigecycline decreased the hemolytic activity, as well as the skin abscess formation and bacterial burden in mice. Most importantly, it significantly decreased the expression of *hla*, *hlgB*, *hlgC*, *spa*, *sbi*, *saeR*, *sak*, *tst*, and *coa* genes by RT-qPCR and the protein expression of α-toxin. Altogether, the sub-MICs of tigecycline might be a promising agent to attenuate the virulence of *S. aureus* and its host immune response by inhibiting the SaeRS two-component system and the product of α-toxin.

**IMPORTANCE:**

In this study, the sub-MICs of tigecycline decreased the resistance of *S. aureus* to oxidants and human whole blood. Moreover, tigecycline weakened the cell adhesion level of *S. aureus* and skin abscess formation in mice by reducing bacterial burden. Remarkably, tigecycline decreased the hemolytic activity and significantly downregulated the expression of various virulence genes and α-toxin. This research highlighted that the sub-MICs of tigecycline might be a promising agent to attenuate the virulence of *S. aureus* by inhibiting the product of α-toxin.

## INTRODUCTION

*S. aureus* is a common pathogen that is connected with bloodstream infections, pneumonia, keratitis, osteomyelitis, endocarditis, and skin and soft tissue infections (1–3). The widespread prevalence of antibiotic resistance restrained clinical options for the therapy of *S. aureus* infections (4–6). Especially, the emergence of methicillin-resistant *Staphylococcus maureus* (MRSA) posed a burden on public health (7, 8). Therefore, there was an urgent need to develop and ascertain new antibacterial agents based on virulence factor therapy for the treatment of *S. aureus*. In *S. aureus*, the SaeRS two-component system (TCS) plays an important role in controlling the production of various virulence factors, including α-hemolysin (*hla*), γ-hemolysin (*hlgCB*), surface protein (*spa*), IgG-binding protein (*sbi*), staphylokinase (*sak*), toxic shock toxin (*tst*), and coagulase (*coa*) (9–11). *hla* and *hlgCB* could encode secreted pore-forming toxins that can damage the host cell membrane, one of which is red blood cells (12–15). *spa* is an important cell wall-related virulence factor in *S. aureus*, which can interact with various host immunoglobulins (16, 17). *sbi* is a multifunctional immune evasion factor of *S. aureus* that promotes bacterial survival in whole human blood and the avoidance of neutrophil-mediated opsonophagocytosis (18, 19). *sak* is a fibrinolytic agent that exerts its fibrinolytic function in specific hosts and could also evade innate immunity defenses (20, 21). *tst* can encode thermogenic toxins, and their release into the bloodstream can lead to toxic shock syndrome (22, 23). The function of *coa* is to convert fibrinogen into fibrin, which assists *S. aureus* to evade immune phagocytosis (24, 25). Therefore, the development and application of agents targeting the SaeRS two-component system and virulence genes were also a potential way to treat *S. aureus* infection.

Tigecycline was a 9-t-butylamine derivative of dimethylamine tetracycline and the first drug of glycyrrhizin antibiotics (26). Tigecycline exerted its effect by inhibiting bacterial protein synthesis. As a member of the third-generation tetracycline, tigecycline was approved by the Food and Drug Administration for clinical use in complex abdominal infections, soft tissue infections, and complex skin infections in 2005 (27). The clinical application was launched in China in 2010. This drug was considered the last-line treatment option for complex infections caused by extensively resistant gram-negative and multidrug-resistant gram-positive bacteria (28). Especially, tigecycline is one of the most effective drugs for treating MRSA clinically. Equally important, the effects of tigecycline on liver and kidney function and bone marrow were significantly lower than those of other drugs utilized to treat *S. aureus*, such as vancomycin, teicoplanin, and linezolid (29). Previous research has found that virulence expression would decrease upon treatment with ribosomally active antibiotics (linezolid and clindamycin) (30). Tigecycline inhibits protein translation in bacteria by binding to the 30S ribosomal subunit and blocking the elongation of peptide chains. The blockage of ribosomal translation seems to overshadow the *sae* activation.

The innate immune system was the first line of defense against the invasion of pathogenic microorganisms; however, pathogenic microorganisms had already evolved some immune escape strategies to overcome the body’s immune attacks to survive on their own, causing colonization and infection (31–33). Macrophages are a crucial component of the innate immune system in the human body, capable of recognizing, phagocytosing, and eliminating pathogens and foreign objects (34–36). Most importantly, recent studies have confirmed that once engulfed by macrophages, a certain proportion of *S. aureus* could still survive and even spread (37–39). To inhibit the intracellular survival of invading pathogens, the inflammatory signaling pathway promotes the expression of various pro-inflammatory cytokines, including tumor necrosis factor α (TNFα), interleukin 1β (IL-1β), IL-6, and IL-8 (40–45). Accordingly, it was particularly essential to clarify the effect and specific mechanisms of tigecycline on the counteraction of *S. aureus* against macrophage phagocytosis.

In the present study, we chose two *S*. *aureus* strains, named SA75 and JP30, to investigate the effect of tigecycline on the virulence of *S. aureus*. Since SA75 and JP30 have remarkable virulence, especially hemolytic activity and the ability of mouse skin abscess formation (46, 47), we used them to study the effects of tigecycline on α-toxin. This research is geared toward demonstrating that tigecycline can attenuate the virulence of *S. aureus* and its host immune response by inhibiting the SaeRS two-component system and the product of α-toxin. Tigecycline could become useful as an anti-Hla drug if our results are confirmed by future studies.

## MATERIALS AND METHODS

### Bacterial strains, reagents, cells, and culture conditions

The bacterial strains used in the study were SA75 and JP30. The strain information was shown in Table 1. SA75 (MSSA) was a clinical *S. aureus* strain isolated from patients with suppurative skin infection. JP30 (MRSA) was isolated from the patient’s sputum at the First Affiliated Hospital of Wenzhou Medical University. All strains were incubated in trypticase soy broth (TSB) medium at 37°C with shaking at 220 rpm. Tigecycline (MCE) was purchased from MedChemExpress company and dissolved in dimethyl sulfoxide (DMSO) as a stock solution (200 µg/mL). The A549 lung epithelial cell and RAW264.7 murine macrophage-like cell line were grown in Dulbecco's Modified Eagle Medium (DMEM) medium after supplementing with 10% fetal bovine serum at 37°C and 5% CO_2_.

**TABLE 1:** Bacterial strains that were used in this study

Strain	Tigecycline	Source	Antibiotic resistance/susceptibility profiles
SA75	0.125	Pus	OXA^*a*^(S); TET^*b*^(S); ERY^*c*^(S); VAN^*d*^(S); TEC^*e*^(S); LZD^*f*^(S)
JP30	0.25	Sputum	OXA(R); TET(R); ERY(R); VAN(S); TEC(S); LZD(S)

^*a*^ Oxacillin.

^*b*^ Tetracycline.

^*c*^ Erythromycin.

^*d*^ Vancomycin.

^*e*^ Teicoplanin.

^*f*^ Linezolid.

### Determination of minimum inhibitory concentration

Tigecycline was prepared in DMSO (Biosharp, Beijing, China) at a concentration of 100 µg/mL. The broth microdilution method based on the Clinical and Laboratory Standards Institute (CLSI) guidelines was used to determine the MIC (CLSI, 2019). The colonies were cultured for 16–18 h and directly extracted to prepare a 0.5 MacFarland turbidity standard bacterial suspension, and then diluted with cation-adjusted Mueller–Hinton broth at a 1:100 ratio. A total of 100 µL of medium containing tigecycline (0–0.5 µg/mL) and 100 µl of suspension were added into a 96-well microfilter plate. In the experiment, we used DMSO as a control. After that, the plate was incubated for 16–18 h at 37°C. All assays were performed in triplicate. The minimum concentration at which no bacterial growth was observed by the naked eye was defined as the MIC.

### Growth inhibition assay

*S. aureus* strains were grown in TSB for 4–6 h and made into a bacterial suspension with a turbidity of 0.5 MacFarland standard. Then, we performed 1:100 dilution into the TSB medium containing tigecycline so that the final concentrations of the medium were 0.625–0.015 µg/mL. No drug was added as a positive control; TSB was the negative control. A 200 µL mixed liquid was added to a sterile bioscreen honeycomb plate. We used an automatic microbial growth curve analyzer (OY Growth Curves, Finland) to measure OD600 every 1 h for 24 h and obtain a growth curve according to the measured values. The test was performed in triplicate.

### Checkerboard assay

The checkerboard assay was performed in a 96-well plate. Tigecycline and hydrogen peroxide were adjusted to 2 µg/mL and 1.2 mM, respectively. The antibiotic tigecycline was two-fold serially diluted along the row axis, and the hydrogen peroxide (H_2_O_2_) compound was two-fold serially diluted along the column axis to create a matrix in which each well consists of a combination of both at different concentrations. *S. aureus* (approximately 1.5 × 10^6^ CFU/mL) was transferred into a 96-well plate in a 100 µL final volume. After incubation for 16–20 h at 37°C, the fractional inhibitory concentration (FIC) index was calculated by the summation of both FIC values, which was interpreted as synergistic, additive, or antagonistic for values of *x* ≤ 0.5, 0.5 < *x* < 4, or ≥4, respectively. The FIC index values for H_2_O_2_ and Tigecycline against *S. aureus* SA75 and JP30 were shown in Table 2

**TABLE 2:** FIC index values for H_2_O_2_ and tigecycline against *S. aureus* SA75 and JP30

Isolate	FIC of H_2_O_2_	FIC of tigecycline	FIC index	Interpretation
SA75	0.5	0.25	0.75	Additive
JP30	0.5	0.125	0.625	Additive

### Hydrogen peroxide killing assay

SA75 and JP30 were grown in TSB with or without tigecycline (0.015 µg/mL). Overnight cultures were washed twice with PBS and diluted to a concentration of 1 × 10^6^ CFU/mL. Next, 500 µL of bacterial suspension was added in a 1.5 mL Eppendorf tube. Then, H_2_O_2_ was added to a 1.0 mM final concentration, and the tubes were incubated at 37°C for 1 h with shaking at 220 rpm. The reaction was stopped by the addition of 1,000 U/mL of exogenous catalase (Sigma-Aldrich). Then, the cells were serially diluted with PBS and spread on the trypticase soy agar (TSA) plates. After incubating at 37°C for 24 h, viable cells were counted to assess whether tigecycline affected the sensitivity of *S. aureus* to H_2_O_2_. All tests were run in triplicate.

### Human whole-blood killing assay

SA75 and JP30 were grown in TSB with or without tigecycline (0.015 µg/mL). Overnight cultures were centrifuged at 12,000 rpm for 1 min at room temperature and adjusted to a concentration of 1 × 10^6^ CFU/mL using sterile PBS. The bacterial suspensions were mixed gently with whole blood collected from healthy human volunteers at a ratio of 1:1 in 1.5 mL Eppendorf tubes (1 mL). The tubes were incubated at 37°C for 1 h with shaking (220 rpm), and bacterial viability was determined by plating dilutions on TSA plates. All experiments were run in triplicate.

### Hemolysis assay

The hemolytic activity of strains was measured using human blood. *S. aureus* strains were grown in TSB with or without 0.015 µg/mL tigecycline. After 16 h of incubation, cultures were adjusted to the same optical density (OD600), and then centrifuged at 12,000 rpm for 2 min at room temperature. Then, 100 µL of supernatant was added to 900 µL of phosphate-buffered saline (PBS) with a final concentration of 2% human blood. Blood with Triton X-100 and PBS was used as positive and negative controls, respectively. Subsequently, the samples were incubated at 37°C for 1 h. Next, the mixtures were centrifuged at 6,000 rpm for 5 min, and the absorbance of supernatants was measured at 600 nm. Hemolysis (%) = [(absorbance of the treated sample – absorbance of negative control) / (absorbance of positive control – absorbance of the negative control)] × 100%. All assays were performed in triplicate.

### Cell adhesion assay

The A549 lung epithelial cell was grown in DMEM medium after supplementing with 10% fetal bovine serum at 37°C and 5% CO_2_. About 5 × 10^5^ cells were seeded into 12-well plates. Before use, the plates were washed twice with PBS. SA75 and JP30 were grown in TSB with or without tigecycline (0.015 µg/mL) with shaking for 16 h to the logarithmic growth phase and resuspended in DMEM medium without serum. Then, the cells were infected with bacteria [multiplicity of infection (MOI) = 10:1] and co-incubated at 37°C for 2 h. Subsequently, the supernatant was aspirated and discarded, and the plates were washed three times with sterile PBS to remove loosely adherent bacteria. Then, cells were dissociated with 200 µL trypsin–EDTA for 3 min at 37°C. A549 cells were lysed with 0.05% Triton X-100. Bacterial CFU was determined by plating serial dilutions of epithelial cell lysates onto TSA plates. Each sample was tested in triplicate.

### Assessment of bacterial intracellular survival and product of immune cytokines

The RAW264.7 murine macrophage-like cell line was grown in DMEM medium after supplementing with 10% fetal bovine serum at 37°C and 5% CO_2_. About 1 × 10^5^ cells were seeded into 12-well plates. Before use, the plates were washed twice with PBS. SA75 and JP30 were grown in TSB with or without tigecycline (0.015 µg/mL) with shaking for 16 h to the logarithmic growth phase and resuspended in DMEM medium without serum. Then, the cells were infected with bacteria (MOI = 10:1) and co-incubated at 37°C for 1 h. Following incubation for 1 h, infected cells were washed three times with PBS before the addition of 10% (v/v) FCS–DMEM supplemented with 10 µg/mL lysostaphin (Sigma-Aldrich) and 100 µg/mL gentamicin (Sigma-Aldrich) to each well. Plates were then incubated for 1 h to kill extracellular bacteria. Following incubation, the cells were washed with PBS and further incubated in fresh 10% (v/v) FCS-DMEM. At 0, 4, 8, and 12 h post-infection (hpi), infected cells were washed three times with PBS to remove extracellular bacteria and dead cells and lysed by the addition of 0.5% (v/v) Triton X-100 (Sigma-Aldrich). The number of intracellular bacteria (expressed as colony-forming units, CFU) was determined by serial dilution and plating on TSA agar. RAW264.7 cells (1 × 10^6^ cells/well) in six-well plates were infected with *S. aureus* at an MOI of 10 and incubated at 37°C with 5% CO_2_. At 0, 8, and 12 hpi, the culture medium was removed, and RAW264.7 cells were washed twice with ice-cold PBS. Cell precipitates were collected to subsequently extract RNA.

### Mouse skin abscess model

BALB/C mice (6 weeks old, female) were randomly allocated into five groups (*n*  =  4 per group). SA75 and JP30 were grown in TSB with shaking for 16 h to the logarithmic growth phase and washed three times with PBS. Then, mice were injected subcutaneously into the shaved flank with 100  µL PBS containing 1 × 10^8^ bacterial cells suspended. At 1 h post-infection, PBS containing an equal concentration of DMSO (for controls) or 0.015 µg/mL tigecycline was given directly into the subcutaneous space of the infected area every 24 h for 3 days. The area of skin abscesses was recorded daily using a caliper. Lesion and abscess sizes were monitored daily with a caliper using the formula: (A = π × (L  ×  W)). After measuring and recording the skin abscess for 3 days, the mice were euthanized, and the skin was dissected. The abscess tissue was mixed with 1.0 mL sterile PBS, and then homogenized. Bacterial load was determined in abscess homogenates by serial dilution and culture on TSA plates.

### Real-time fluorescence quantitative PCR

SA75 and JP30 were grown in TSB with or without tigecycline (0.015 µg/mL) for 16 h in TSB with shaking (220 rpm) at 37°C. The total RNA of *S. aureus* was isolated using a Total RNA Purification Kit (Sangon Biotech, Shanghai, China), and the total RNA of RAW264.7 was extracted using Trizol reagent (Invitrogen) by following the manufacturer’s protocol. Total RNA of *S. aureus* and RAW264.7 cells were reverse transcribed into cDNA separately using the PrimeScript RT Reagent Kit with gDNA Eraser (Takara Bio, Inc.). *gyrB* and GAPDH were used as the internal reference genes for *S. aureus* and RAW264.7, respectively. Real-time quantitative PCR (RT-qPCR) was performed using TB GreenTM Premix Ex Taq II (Takara) on QuantStudioTM 5 Real-time PCR System (Applied Biosystems). RNA expression levels of *S. aureus*-related virulence genes, including *hla*, *hlgB*, *hlgC, spa*, *sbi*, *sak*, *tst*, and *coa*, as well as their regulatory gene, *saeR*, and RAW264.7-activated immune cytokines containing IL1β, IL6, IL8, and TNFα genes were calculated by the formula 2^−ΔΔCt^. The primer pairs are shown in Table 3. Each reaction was performed thrice.

**TABLE 3:** Primer pairs used in RT-PCRs

Primer	Primer sequence (5′ → 3′)
Mouse-IL1b-F	GCAACTGTTCCTGAACTCAACT
Mouse-IL1b-R	ATCTTTTGGGGTCCGTCAACT
Mouse-TNFα-F	GAACTGGCAGAAGAGGCACT
Mouse-TNFα-R	CGTGGTGGCCCCTGCCACAAG
Mouse-IL6-F	TAGTCCTTCCTACCCCAATTTCC
Mouse-IL6-R	TTGGTCCTTAGCCACTCCTTC
Mouse-IL8-F	TTGCCTTGACCCTGAAGCCCCC
Mouse-IL8-R	GGCACATCAGGTACGATCCAGGC
Mouse-GAPDH-F	AGGTCGGTGTGAACGGATTTG
Mouse-GAPDH-R	TGTAGACCATGTAGTTGAGGTCA
*hla*-F	AGCGAAGTCTGGTGAAA
*hla*-R	AACTAGAAATGGCTCTATGA
*hlgB*-F	GGCAGACAAAGCAGTGCATA
*hlgB*-R	TTAGCGCCATCTTGTCTGTG
*hlgC*-F	ATTTCCAATCAGCCCCATCACTCG
*hlgC*-R	CAAGAGGTGGTAACTCACTGTCTGG
*spa*-F	AAGGCTAATGATAATCCACC
*spa*-R	GATAAGAAGCAACCAGCA
*sbi*-F	ACAGCAAGAACCCAGAC
*sbi*-R	TGTTGGCTTCTATCAGG
*saeR*-F	TTCACGGTATTAGCATCT
*saeR*-R	GGTCACGAAGTCCCTAT
*sak*-F	GATCTTTGCGCTTGGAT
*sak*-R	AAACCTGGGACTACACTT
*tst*-F	CGAGTCCTTATTATAGCCCTGC
*tst*-R	GTTCCTTCGCTAGTATGTTGGC
*coa*-F	AAATTCCACAGGGCACA
*coa*-R	CGGGACCTTGAACGATT
*gyrb*-F	ACATTACAGCAGCGTATTAG
*gyrb*-R	CTCATAGTGATAGGAGTCTTCT

### Western blot analysis

Western blots were performed as previously described (48). Briefly, SA75 and JP30 were grown in TSB with or without tigecycline (0.015 µg/mL) with shaking for 16 h, and bacterial culture supernatants were collected. The protein concentration of each sample was determined using the Bradford assays to maintain consistency in the total amount of proteins loaded by SDS-PAGE among all the strains. The protein was mixed with Omni-Easy Protein Sample Loading Buffer (EpiZyme Biotechnology Co., Ltd., Shanghai, China), and the mixture was denatured at 95°C for 10 min. The samples were separated by 12.5% SDS-PAGE and transferred onto a polyvinylidene fluoride membrane. After blocking with 5% bovine serum albumin at room temperature for 3 h, the membrane was incubated overnight with a rabbit-derived primary anti-Hla IgG antibody (Sigma) at a dilution of 1/2,500. Subsequently, the membrane was incubated with a goat-derived secondary anti-rabbit antibody (Biosharp) at room temperature for 2 h at a dilution of 1/5,000. The Omni ECLTM Pico Photoluminescence Kit (EpiZyme Biotechnology Co., Ltd., Shanghai, China) was used for developing images, and the ImageJ software was used to analyze these images.

### Statistical analysis

All analyses were performed using GraphPad Prism 8 (GraphPad Software, Inc., San Diego, CA, USA). Results derived from samples between two groups were treated with unpaired two-tailed Student’s *t*-test and *χ* test. *P* < 0.05 was statistically considered to be significant. *P < 0.05, **P < 0.01, ***P < 0.001, and ****P < 0.0001.

## RESULTS

### Influence of subinhibitory concentrations of tigecycline on the growth of *S. aureus* strains

**Fig 1:**
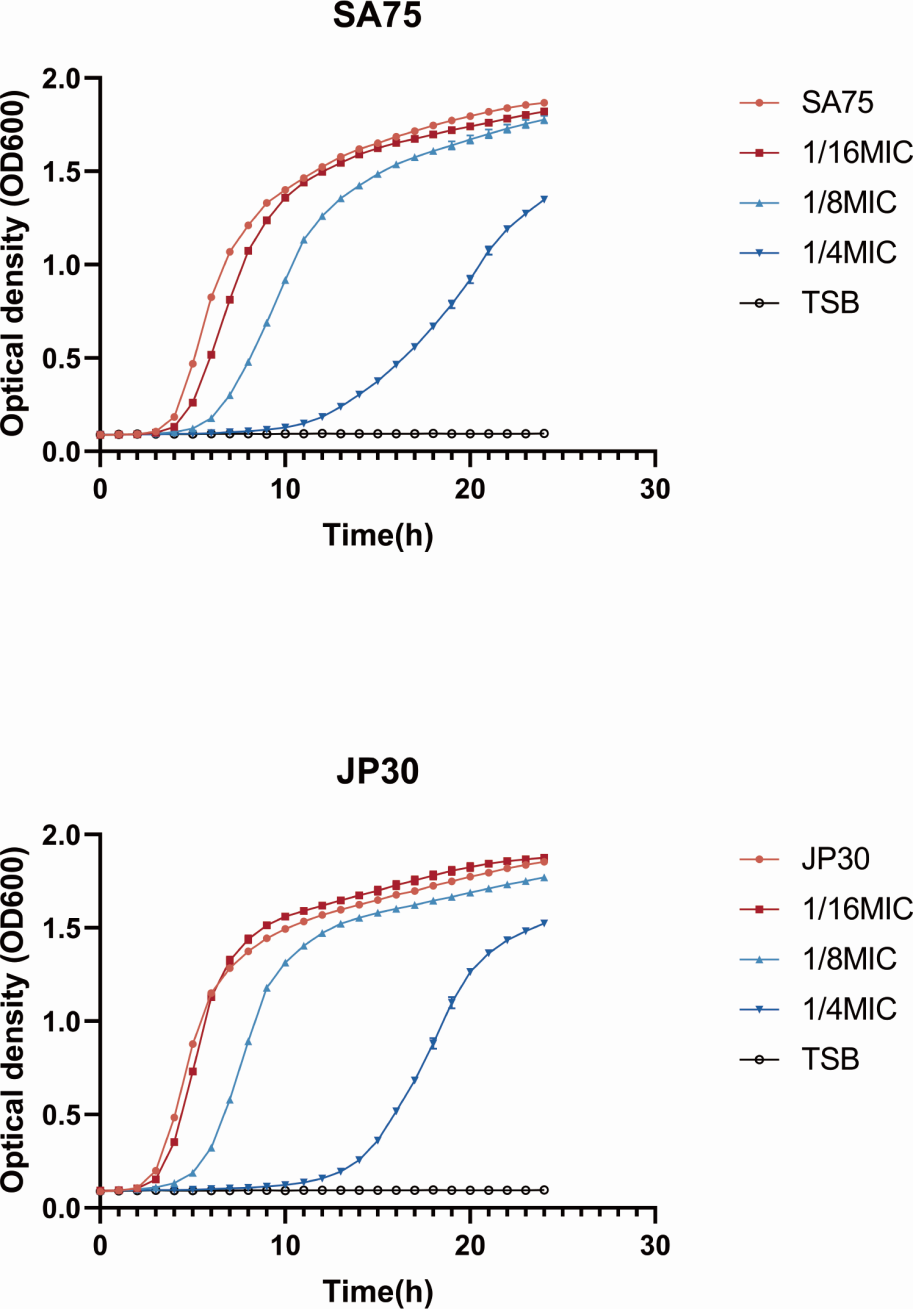
Influence of subinhibitory-MICs of tigecycline on the growth of *S. aureus* SA75 and JP30 strains. TSB was used as a blank control.

The MIC values of tigecycline against SA75 and JP30 were 0.125 and 0.25 µg/mL, respectively. We selected the minimum concentration of tigecycline that could inhibit the virulence of *S. aureus* without affecting the growth of the bacteria. At the subinhibitory concentration of 0.015 µg/mL, the OD of bacteria at the late logarithmic growth period was consistent (Fig. 1). Thus, we chose the concentration of 0.015 µg/mL for the experiment, which is 1/8 MIC for SA75 and 1/16 MIC for JP30.

### Tigecycline sensitized *S. aureus* to H_2_O_2_ and human whole blood

**Fig 2:**
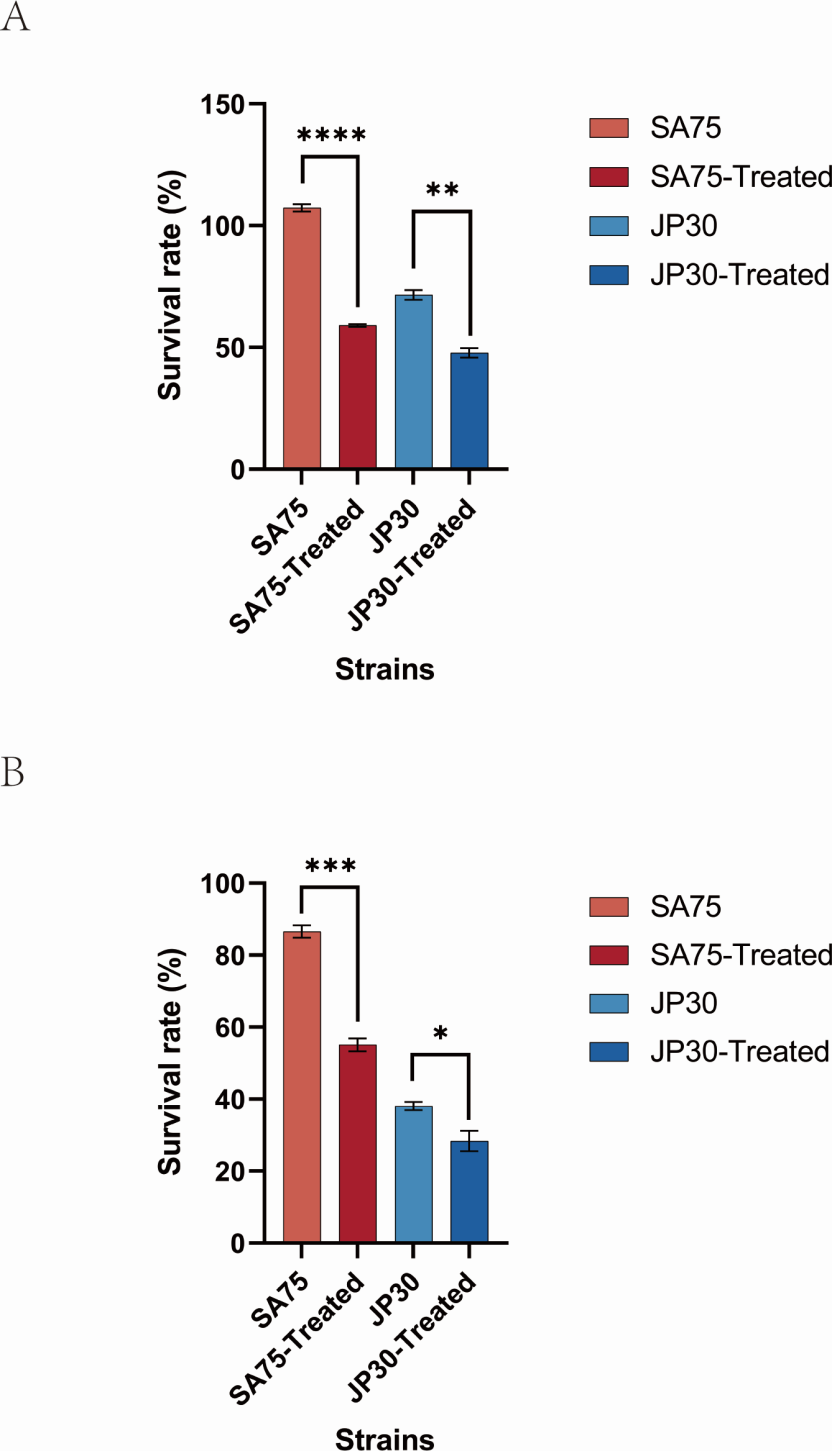
Effect of tigecycline on the susceptibility of *S. aureus* SA75 and JP30 to killing by either H_2_O_2_ (**A**) or human whole blood (**B**). Error bars in the figures represent the standard deviation of a data set (mean  ±  standard). *P < 0.05, **P < 0.01, ***P < 0.001, and ****P < 0.0001.

We compared the sensitivities of tigecycline-treated and untreated *S. aureus* to H_2_O_2_. The MIC of H_2_O_2_ was 0.35 mM, and we selected a concentration of H_2_O_2_ (1.0 mM) that had a suitable killing effect on *S. aureus*. Survival of the tigecycline-treated SA75 was lower than that of the untreated SA75 (59.03% ± 1.0% vs. 107.4% ± 2.6%; *P* < 0.0001). Notably, survival of the tigecycline-treated JP30 was lower than that of the untreated JP30 (47.75% ± 3.4% vs. 71.54% ± 3.4%; *P* < 0.01) (Fig. 2A). Subsequently, we investigated whether tigecycline sensitizes *S. aureus* to human whole blood. As shown in Fig. 2B, the survival of tigecycline-treated SA75 was lower than that of the untreated SA75 (55.12% ± 3.1% vs. 86.58% ± 2.9%; *P* < 0.001) and that of tigecycline-treated JP30 compared with the untreated JP30 (28.37% ± 4.9% vs. 38.07% ± 1.9%; *P* < 0.05). The human whole-blood killing assay suggested that a part of *S. aureus* was sensitive to human blood immune clearance after tigecycline treatment.

### Tigecycline weakened the cell adhesion level of *S. aureus*

**Fig 3:**
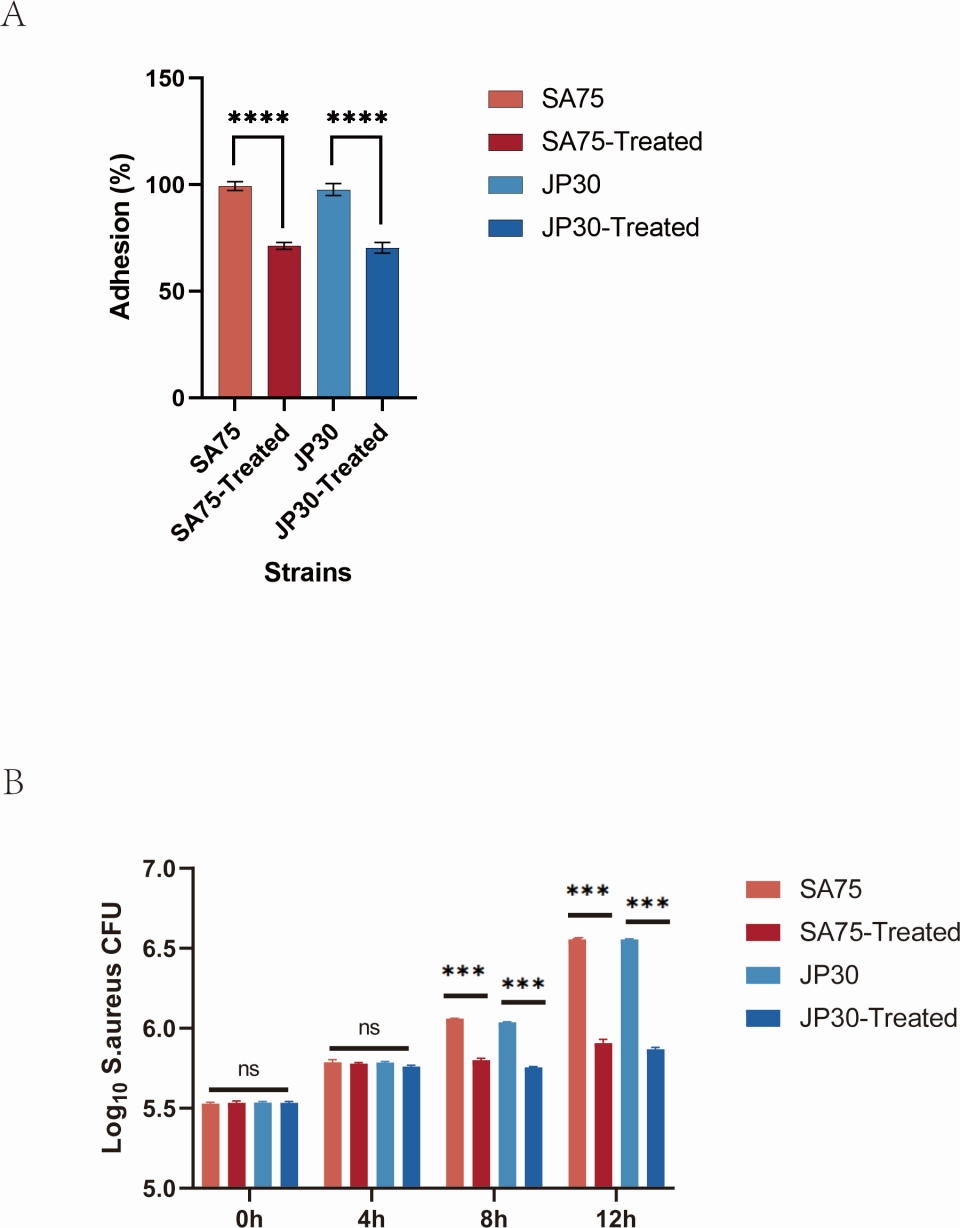
Effect of tigecycline on the ability of cell adhesion in A549 cell (**A**) and the survival rate in RAW264.7 cell (**B**). Error bars in the figures represent the standard deviation of a data set (mean  ±  standard). ***P < 0.001 and ****P < 0.0001.

To further explore the influence of tigecycline on the cell adhesion capabilities of *S. aureus*, we performed the cell adhesion assay with or without tigecycline. As shown in Fig. 3A, treatment with tigecycline significantly reduced the A549 epithelial cell adhesion ability of SA75 and JP30. Tigecycline-treated SA75 exhibited markedly decreased cell adhesion compared to untreated SA75 (71.35% ± 3.3% vs. 99.38% ± 4.2%; *P* < 0.0001). Consistent with that, JP30 treated with tigecycline represented a remarkable decrease in adhesion in comparison with untreated JP30 (70.45% ± 6.2% vs. 97.74% ± 6.8%; *P* < 0.0001).

### Tigecycline undermined the survival of *S. aureus* in RAW264.7

The effect of tigecycline on *S. aureus* to persist intracellularly was quantified using a lysostaphin/gentamicin protection assay in RAW264.7 cells. We infected RAW264.7 with the bacterial suspension. Following incubation for 1 h, infected cells were washed with PBS before the addition of lysostaphin and gentamicin to kill extracellular bacteria. Further incubated until 0, 4, 8, and 12 hpi, the CFU of intracellular bacteria was determined and plated on TSA agar. We observed that there were no differences in survival between the tigecycline-treated group and the untreated SA75 and JP30 group at 4 hpi. Nonetheless, the tigecycline-treated group showed noticeably decreased survival rates in RAW264.7 at 8 and 12 hpi (Fig. 3B). Survival of the tigecycline-treated SA75 and JP30 in RAW264.7 was decreased by ~0.26 [*P* < 0.0001; 95% confidence interval (CI), −0.2942 to −0.2264] and ~0.28 (*P* < 0.0001; 95% CI, −0.2999 to −0.2632) log ^10^ CFU/well, respectively, at 8 hpi. Notably, the survivals of the tigecycline-treated SA75 and JP30 strains in RAW264.7 were decreased by ~0.65 (*P* < 0.0001; 95% CI, −0.7169 to −0.5813) and ~0.69 (*P* < 0.0001; 95% CI, −0.7205 to −0.6518) log ^10^ CFU/well separately at 12 hpi. The above outcomes revealed that tigecycline weakened the ability of *S. aureus* to resist macrophage phagocytosis and degradation.

### Tigecycline attenuated macrophage inflammatory response induced by *S. aureus*

**Fig 4:**
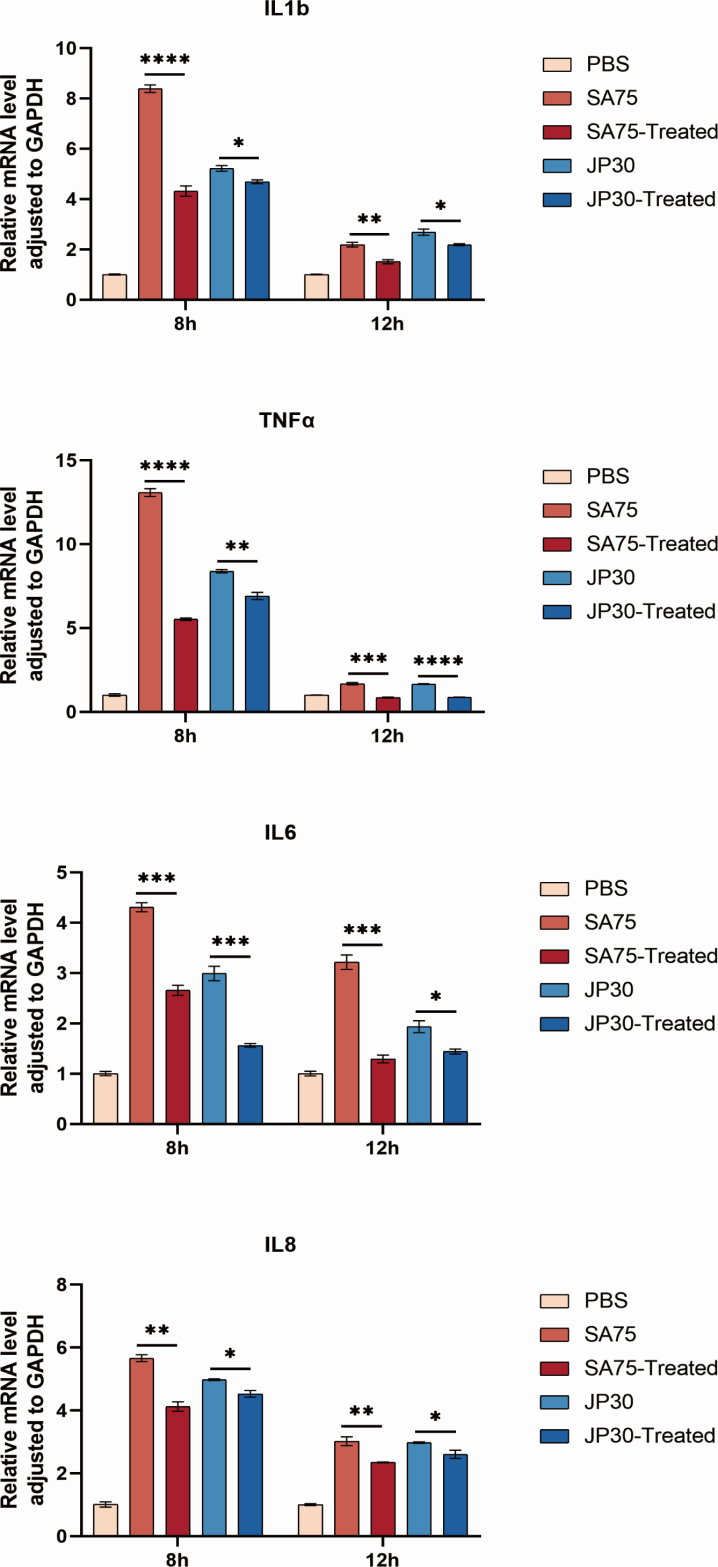
Effect of tigecycline on the inflammatory response in RAW264.7 cell. The transcript levels of immune cytokines, including IL1β, IL6, IL8, and TNFα genes, were normalized to the GAPDH transcript level. *P < 0.05, **P < 0.01, ***P < 0.001, and ****P < 0.0001.

To inhibit the intracellular survival of invading pathogens, the inflammatory signaling pathway promotes the expression of various pro-inflammatory cytokines, including TNFα, IL1β, IL6, and IL8. We collected the cell precipitation and isolated the RNA of RAW264.7 at 8 and 12 hpi exclusively for the reason that there were only significant differences between the tigecycline-treated and untreated SA75 and JP30 groups at 8 and 12 hpi. The transcript levels of immune cytokines were determined using RT-qPCR to clarify the effect of tigecycline on the inflammatory response induced by *S. aureus*. In general, the results showed that in RAW264.7, the expressions of IL1β, IL6, IL8, and TNFα genes were altogether downregulated considerably to varying degrees with the treatment of tigecycline regardless of whether it was 8 or 12 hpi (Fig. 4). As a corollary, these findings indicated that tigecycline inhibited macrophage pro-inflammatory response induced by *S. aureus*.

### Tigecycline effectively reduced the formation of skin abscesses in mice after *S. aureus* infection

**Fig 5:**
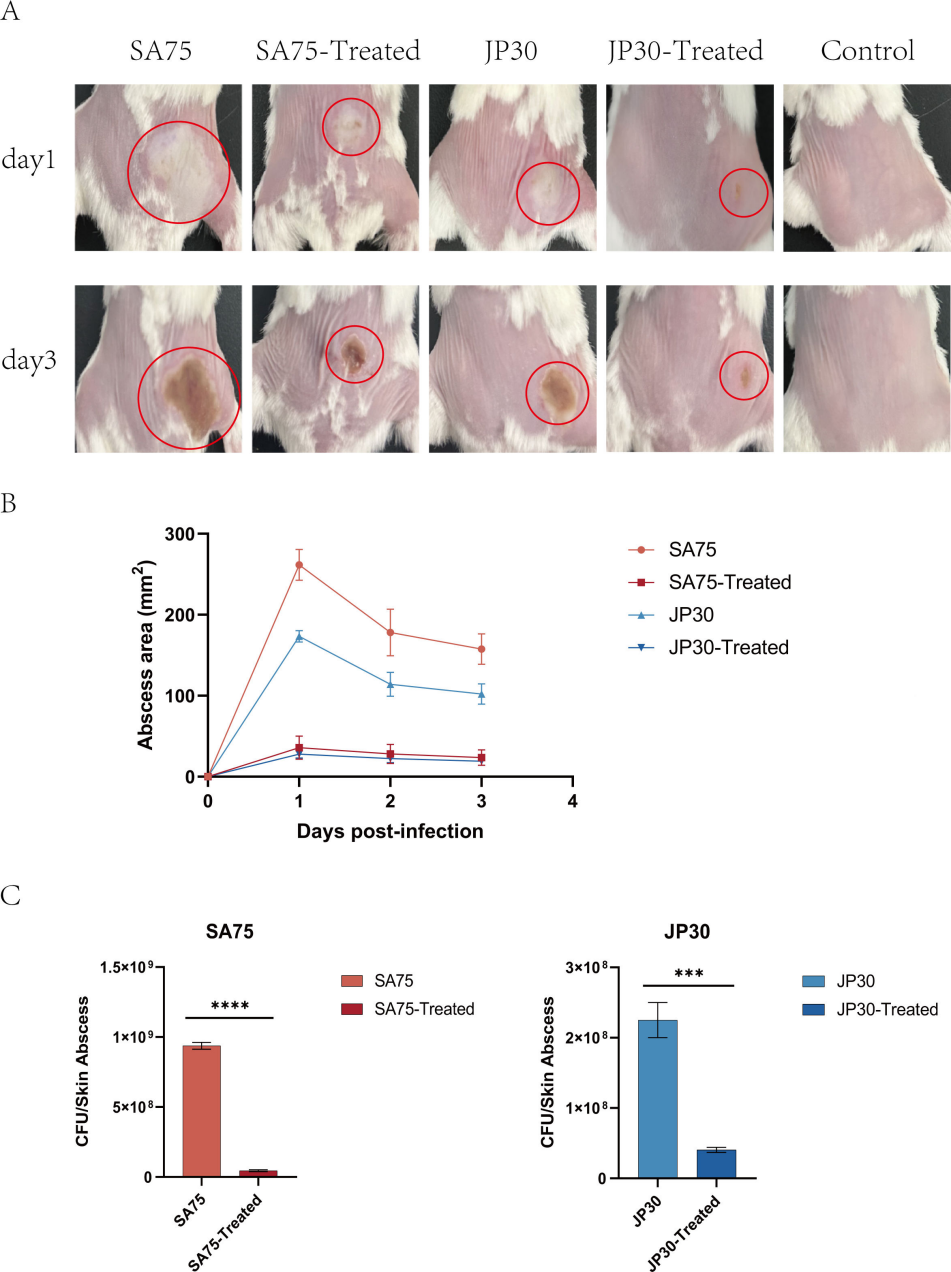
Tigecycline effectively reduced the formation of skin abscesses in mice after *S. aureus* infection. (**A**) The image of representative abscesses before and after tigecycline-treated strains at days 1 and 3 after infection. (**B**) Daily changes of the skin abscess area in mice before and after treatment. (**C**) Comparison of bacterial colonies in mice skin of *S. aureus* wild-type group and *S. aureus* treated group. ***P < 0.001 and ****P < 0.0001.

We used a mouse skin abscess model to further explore the effects of tigecycline on skin abscess formation and invasiveness of *S. aureus in vivo*. We injected 100  µL PBS containing 1 × 10^8^ bacterial cells subcutaneously into the back of mice, and the amount of injection was the same in each mouse. The sizes of abscesses formed after infection were different. We tried to choose the same place, and the area of abscess formation was easy to observe. As shown in Fig. 5A, treatment with tigecycline significantly restrained the size of abscesses after 1 day of infection and significantly reduced the formation of yellow eschar on day 3. Figure 5B plotted changes in the abscesses area of SA75 and JP30 before and after treatment with tigecycline, respectively. Abscesses in all groups were most pronounced on day 1 after infection, and then began to decrease and heal gradually. Notably, the abscess sizes of the SA75 wild-type group were significantly larger than those of the SA75-treated group on the first day post-infection (261.51 mm^2^ vs. 35.87 mm^2^). Mice were sacrificed 4 days after infection, and bacterial load in the skin was determined. After treatment, the loads of SA75 and JP30 were decreased significantly by 82 and 95.2%, respectively (Fig. 5C, left). The above findings showed that tigecycline could weaken the virulence of *S. aureus*.

### Tigecycline downregulated the expression of virulence-related genes of *S. aureus*

**Fig 6:**
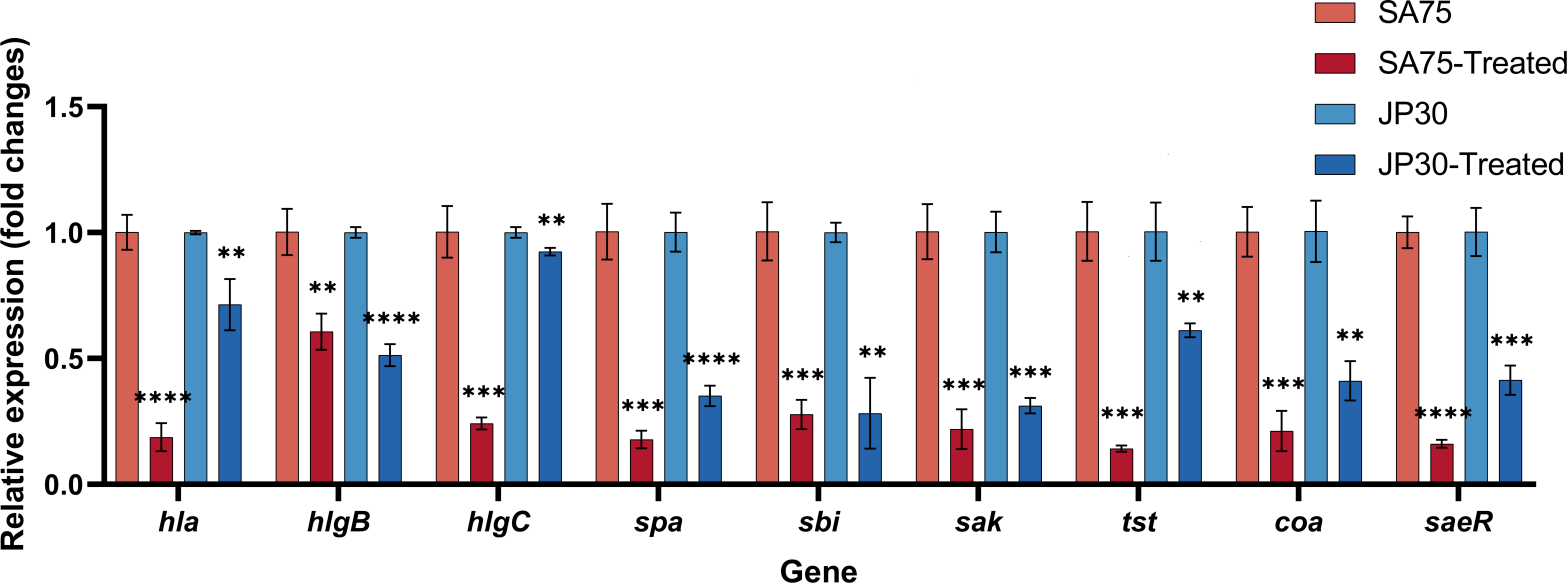
Effect of tigecycline on the expression of virulence-related genes in *S. aureus*. Transcript levels were normalized to the *gyrB* transcript level. **P < 0.01, ***P < 0.001, and ****P < 0.0001.

The transcript levels of virulence-related genes treated with the concentration of 0.015 µg/mL of tigecycline were determined using RT-qPCR to clarify the effect of tigecycline on the virulence of *S. aureus* strains. In general, the results showed that in SA75 and JP30, the expressions of *hla*, *hlgB*, *hlgC, spa*, *sbi*, *saeR*, *sak*, *tst*, and *coa* genes were remarkably downregulated to varying degrees with the treatment of tigecycline (Fig. 6). Particularly, the expressions of *hla* and *saeR* in the SA75-treated group were significantly decreased by 81 and 84%, respectively. The above results suggested that tigecycline might affect the hemolytic ability of *S. aureus*.

### Tigecycline decreased the hemolytic activity and the α-toxin protein level of *S. aureus*

**Fig 7:**
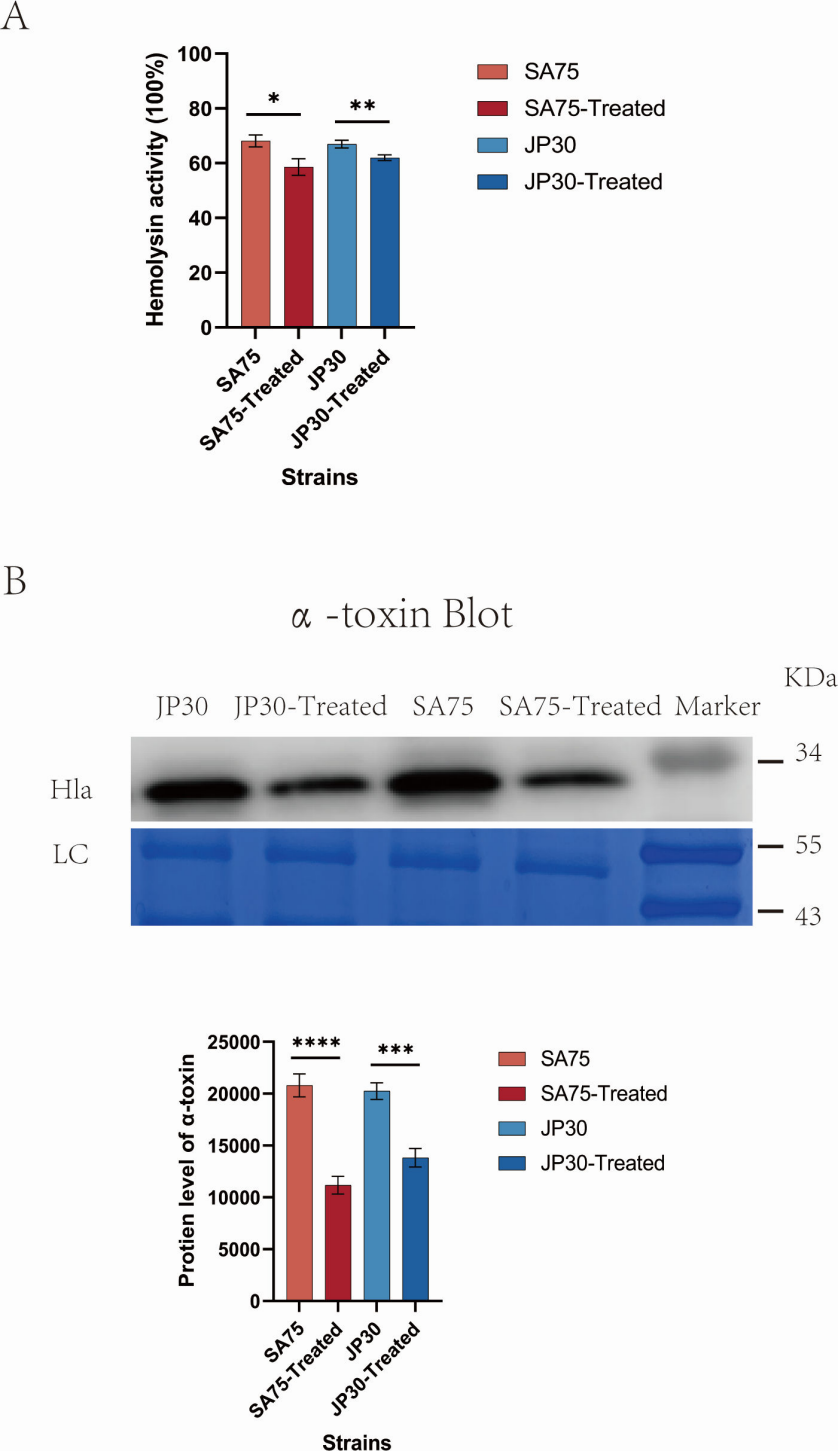
Effect of tigecycline on the hemolytic activity (**A**) and the protein level of α-toxin (**B**). The total bacterial proteins served as loading control (LC). Molecular weights of the protein marker were indicated on the left. The gray values of the secretion of α-toxin were measured by ImageJ software. Error bars in the figures represent the standard deviation of a data set (mean  ±  standard). *P < 0.05, **P < 0.01, ***P < 0.001, and ****P < 0.0001.

The effect of tigecycline on hemolysin in *S. aureus* culture supernatants was assessed using hemolysin assays, with the percentage of hemolysis obtained by comparison with the untreated group. Tigecycline treatment depressed the hemolysis activity in culture supernatants of SA75 and JP30 (Fig. 7A). This might be explained by the downregulation of the expressions of virulence genes *hla* and *hlgCB* in bacteria after treatment with tigecycline (Fig. 6). We next tested whether tigecycline exhibited high anti-virulence activity due to high *hla* expression by western blot. A significant difference in the protein expression level of α-toxin between *S. aureus* and tigecycline-treated *S. aureus* was observed (Fig. 7B). Compared to untreated SA75, tigecycline-treated SA75 exhibited significantly reduced α-toxin levels (11,170 ± 2,094 vs. 20,796 ± 2,707; *P* < 0.0001). In line with this, when compared with untreated JP30, the α-toxin level in tigecycline-treated JP30 was notably decreased (13,817 ± 892.3 vs. 20,258 ± 802.3; *P* < 0.001). Sub-MICs of tigecycline displayed an anti-toxin effect on Hla expression. These results were consistent with the above virulence phenotype experiments. Given that the SaeRS two-component system could regulate the expression of the abovementioned seven genes, except for itself, we would be correct to assume that tigecycline might inhibit the SaeRS two-component system to reduce the mRNA and the protein expression of *hla* and finally weaken the virulence of *S. aureus*.

## DISCUSSION

*S. aureus* mainly tended to cause skin and soft tissue infections, and its pathogenicity was demonstrated by producing multiple virulence factors (49). The inhibiting toxin of *S. aureus* was a potential target for antibacterial therapy (50–52). The antibacterial mechanism of tigecycline is to inhibit bacterial growth by binding to the 30S ribosomal subunit, preventing the binding of aminoacyl tRNA to bacterial ribosomes (53). Atshan et al. suggested that sub-MIC usage of tigecycline could signal virulence induction by *S. aureus* via the regulation of biofilm adhesion factor genes and exoproteins (54). Smith et al. highlighted that tigecycline altered the expression of *tst*, which encoded toxic shock syndrome toxin 1 considered to be crucial for the virulence of *S. aureus* (55). The above research emphasized the considerable role of sub-MICs of tigecycline in modulating virulence in *S. aureus*.

In S. *aureus*, SaeRS TCS played a crucial role in controlling the production of over 20 virulence factors, including hemolysin, superantigens, surface proteins, coagulase, and so forth (56–59). SaeRS TCS is composed of a sensor histidine kinase SaeS, a response regulatory factor SaeR, and two co-proteins, SaeP and SaeQ (11). Since its discovery in 1994, the *sae* locus has been studied extensively, and its contribution to the virulence and pathogenesis of *S. aureus* has been well documented and understood. Gudeta et al. revealed a drastic reduction of α-toxin levels in the culture supernatants of SaeR-binding mutant in contrast to the marked α-toxin production in the wild type, which indicated there was a direct effect of *hla* regulation by SaeR on pathogenesis (60). Rogasch et al. found that a loss of SaeRS resulted in a decreased amount of at least 17 extracellular proteins, among them important virulence factors, such as *hlgCB* (61). Voyich et al. discovered that the expression of *sbi* could be regulated by SaeRS during *S. aureus* evasion of human polymorphonuclear leukocyte killing, and the expression of *sak* was down-regulated in the *saeR*/*S* mutant strain (10). Baroja et al. demonstrated that the SaeRS TCS was a positive transcriptional regulator that bound directly to the *tst* promoter, and SaeR was required for the expression of *tst* (9). Mainiero et al. clarified that SaeR was necessary for the activation of class I target genes *coa* (62). Accordant with a previous study (63), our findings revealed that sub-MICs of tigecycline significantly decreased the mRNA expression of *hla* and the protein expression of α-toxin.

*S. aureus* usually causes skin infections (64–66). As shown in the mouse skin abscess model, we demonstrated the robust efficacy of tigecycline against *S. aureus in vivo*. The skin abscess area and bacterial burden in JP30 and SA75 infected mice were significantly improved by tigecycline. The decreased bacterial population of skin abscesses pointed out that reduced virulence in *S. aureus in vivo* might contribute to its decreased immune evasion ability. Surprisingly, tigecycline also had an obvious curative effect on SA75 with a larger skin infection area and more bacterial burden. Apart from that, we indicated that tigecycline enhanced the sensitivity of *S. aureus* to oxidants and human whole blood. These further confirmed that tigecycline could weaken the virulence of *S. aureus*.

Previous investigations suggested the SaeRS TCS was essential for innate immune evasion by *S. aureus* (10, 67–69). The pathogenesis of *S. aureus* was closely related to its ability to directly adhere to host cells or extracellular matrix. Bacterial adhesion was the first step in invading host cells (70–72). Furthermore, *S. aureus* could invade and multiply in immune cells, such as macrophages (73–75). Gardete et al. believed that under the action of antibiotics, *S. aureus* could regulate the expression of virulence factors to escape host immune attacks and finally contribute to its survival (76–78). In our experiment, sub-MICs of tigecycline weakened the cell adhesion level of *S. aureus*. Zhang et al. demonstrated that intracellular *L. monocytogenes* hijacked autophagy in macrophages by secreting the virulence factor listeriolysin O to evade killing (79). Dai et al. revealed that heterogeneous vancomycin-intermediate *S. aureus* used the vraSR regulatory system to modulate autophagy for increased intracellular survival in macrophage-like cell line RAW264.7 (80). Consistent with the above research, we authenticated that sub-MICs of tigecycline undermined the survival of *S. aureus* in RAW264.7 macrophages by inhibiting the SaeRS two-component system and the product of α-toxin. In addition, the expressions of IL1β, IL6, IL8, and TNFα genes were altogether downregulated considerably to varying degrees with the treatment of tigecycline, proving that sub-MICs of tigecycline could attenuate macrophage inflammatory response induced by *S. aureus*.

To summarize, our work highlighted that sub-MICs of tigecycline might be a promising agent to attenuate the virulence of *S. aureus* and its host immune response by inhibiting the SaeRS two-component system and the product of α-toxin. We provided novel insights into the anti-virulence of sub-MICs of tigecycline in *S. aureus*, which might be more beneficial for the treatment of *S. aureus* infection.
